# Neural activation during processing of emotional faces as a function of resilience in adolescents

**DOI:** 10.1007/s00787-025-02703-y

**Published:** 2025-04-10

**Authors:** Steve Eaton, Harriet Cornwell, Jack Rogers, Stephane De Brito, Nicola Toschi, Christina Stadler, Nora Raschle, Kerstin Konrad, Gregor Kohls, Areti Smaragdi, Karen Gonzalez-Madruga, Maaike Oosterling, Anne Martinelli, Anka Bernhard, Christine M. Freitag, Catherine Hamilton-Giachritsis, Graeme Fairchild

**Affiliations:** 1 Department of Psychology, University of Bath, 10 West, Bath, BA2 7AY UK; 2 School of Psychology, University of Birmingham, Birmingham, UK; 3 Department of Biomedicine and Prevention, University of Rome ‘Tor Vergata’, Rome, Italy; 4 Jacobs Center for Productive Youth Development, University of Zurich, Zurich, Switzerland; 5 Child Neuropsychology Section, Department of Child and Adolescent Psychiatry, Psychosomatics, and Psychotherapy, University Hospital RWTH, Aachen, Germany; 6 Department of Child and Adolescent Psychiatry, Faculty of Medicine, TUD Dresden University of Technology, German Center for Child and Adolescent Health (DZKJ), Partner site Leipzig/Dresden, Dresden, Germany; 7 Child Development Institute, Toronto, Canada; 8 Department of Child and Adolescent Psychiatry, King’s College London, London, UK; 9 Department of Child and Adolescent Psychiatry, Psychosomatics, and Psychotherapy, University Hospital Frankfurt, Goethe University Frankfurt, Frankfurt Am Main, Germany; 10 School of Psychology, Fresenius University of Applied Sciences, Frankfurt Am Main, Germany

**Keywords:** Resilience, Adversity, fMRI, Youth, Face processing, Emotion

## Abstract

Neuroimaging studies suggest that resilience to adversity is linked to reduced emotional reactivity or enhanced emotion regulation. However, such studies are scarce and mainly use adult samples and categorical definitions of resilience. Using a novel, data-driven approach to define resilience dimensionally, based on cumulative adversity exposure across childhood and psychopathology, we investigated associations between resilience and brain activation during facial emotion processing in youth. We also tested for sex differences in the relationship between resilience and brain activation. fMRI data were acquired from 208 youths (aged 9–18 years; Mean age = 13.28), while viewing angry, fearful, and neutral faces. Whole-brain analyses were performed, followed by region-of-interest analyses focusing on the amygdala, hippocampus, and prefrontal cortex. Resilience was positively correlated with bilateral inferior frontal gyrus responses to fearful (versus neutral) faces, and negatively correlated with right superior temporal gyrus, left hippocampal, and right inferior frontal gyrus responses to neutral faces (versus fixation). Sex-by-resilience interactions were observed in the medial prefrontal cortex: males showed positive, while females showed negative, associations between resilience and brain activation, though these results did not survive correction for multiple comparisons. These findings provide further evidence that resilience in youth is associated with enhanced emotion regulation at a neural level.

**Supplementary Information:**

The online version contains supplementary material available at 10.1007/s00787-025-02703-y.

## Introduction

Childhood adversity is highly prevalent. Up to half of all children and adolescents worldwide experience at least one form of adverse event (e.g., parental divorce), and 10–15% experience more extreme forms of adversity (e.g., maltreatment; [1, 2]). The literature examining the impact of childhood adversity on mental health is consistent in showing that it is predictive of many different forms of psychopathology, including psychosis, internalising disorders such as depression, and externalising disorders such as conduct and oppositional disorders [3–6]. However, despite a robust association between childhood adversity and later psychopathology, many young people who experience adversity do not go on to develop mental health problems and instead demonstrate psychological resilience [7].

Resilience has been defined in various ways [8]. One widely accepted conceptualisation is that it involves positive adaptation (such as positive functioning in social, emotional, and educational or occupational domains) despite exposure to adversity [9]. Accordingly, resilience is defined here as remaining free of mental health problems despite experiences of adversity [10, 11].

Identifying potential psychological processes that promote resilience could inform clinical interventions aimed at minimising the likelihood that an individual will develop psychiatric symptoms following adversity. An increasing body of research has identified various factors that support resilient functioning. These include external factors such as social support [12], and individual factors such as using adaptive emotion regulation strategies (i.e., cognitive reappraisal; [13]).

Using facial emotion processing tasks, neuroimaging studies investigating resilience and emotion processing in adults have shown that resilient individuals show greater activation in brain areas implicated in emotion regulation. For example, Kaldewaij et al. [14] found that adults with fewer post-traumatic stress disorder (PTSD) symptoms following trauma exposure showed greater frontopolar prefrontal cortex activation during an approach-avoidance task when performing affect-incongruent actions (moving a joystick towards angry or away from happy faces). In terms of studies of young people, Wymbs et al. [15] found that maltreated adolescents who exhibited fewer anxiety symptoms (considered ‘resilient’) showed lower amygdala responses to fearful faces than those with elevated anxiety. Despite the heterogeneity in terms of how these studies operationalised resilience, collectively they suggest that resilient individuals show greater prefrontal activation and lower amygdala responses when processing emotional faces. This implies that resilient individuals are better at regulating their emotional responses to negative stimuli or are less reactive to such stimuli [16].

Nevertheless, there are few fMRI studies of resilience in youth, so much of our understanding of the neurobiological basis of resilience is based on evidence from adult samples. Given that many mental health problems emerge during childhood and adolescence, and childhood-onset disorders can be more persistent than adult-onset disorders [17], it is crucial to identify similarities and differences in brain activation between resilient young people and adults. That is, resilient youth and adults may demonstrate similar activation patterns in the same regions, in distinct regions, or in the same regions but in opposite directions. Identifying such similarities and differences could be valuable in the development of clinical interventions that harness resilience strategies in those with a history of adversity or trauma, as they may need to be tailored depending on the patient’s age.

Another important consideration is the definitions of resilience used in previous studies, as these are typically narrow in scope. That is, a common approach consists of treating resilience as an all-or-nothing phenomenon, with individuals categorised as *either* resilient *or* vulnerable based on, for example, whether they remain free of PTSD following childhood abuse. This approach may not fully capture the complexity of resilience. Instead, dimensional measures of resilience can be obtained using a residual regression approach (see [12]). This operationalises resilience by regressing continuous measures of adversity/trauma exposure that capture a wide range of adversities that vary in severity against continuous measures of different forms of psychopathology. Establishing how far an individual deviates from the expected positive, linear relationship between adversity and psychopathology is the key measure of resilience. In other words, those who were lower than expected in terms of psychopathology based on their level of adversity exposure had higher resilience scores, whereas those higher in psychopathology than expected given their level of adversity exposure had lower resilience scores. This approach to measuring resilience may be more valid and powerful than comparing groups using a categorical approach, as these classify individuals based on arbitrary thresholds [18].

A further area for investigation has been highlighted by several reviews (e.g., [19–21]) which suggest that the (neural) mechanisms underlying resilience may differ between sexes. However, very few neuroimaging studies have tested for sex differences in resilience in young people, and none of these employed functional MRI methods [21]. This is important, as compared to males, exposure to adversity during puberty can result in an increased risk for developing mood disorders in females [22], and because females are more than twice as likely to develop PTSD following trauma than males [23]. Therefore, it cannot be assumed that similar brain mechanisms confer resilience in males and females.

To address these gaps in the fMRI literature on resilience, we used a residual regression approach to compute continuous resilience scores in a large, European sample of children and adolescents. We then tested for associations between resilience and brain activation during facial emotion processing, and sex differences in these associations. We utilised a facial emotion processing task that included both emotional and neutral faces, with the latter used to investigate brain activity during face processing in general. Furthermore, we adopted a data-driven, dimensional approach to defining resilience that captures multiple adversities and different forms of psychopathology [24]. We defined adversity as a significant event or stressor, either acute or chronic, that has the potential to disrupt an individual’s normal functioning and necessitates adaptation or ways of coping. Two earlier studies from our laboratory examined grey matter volume [24] and cortical structure [25] markers of resilience in young people using voxel- and surface-based morphometry respectively. These studies found that resilience was positively associated with grey matter volume in the right inferior frontal and medial frontal gyri, and right lateral occipital surface area and right superior frontal gyrification, and negatively associated with left inferior temporal surface area. The current study builds on these findings using functional MRI methods in an overlapping sample.

Based on previous literature and our systematic review of neuroimaging studies of resilience in youth [21], we expected to observe negative associations between resilience and amygdala responses, and positive associations between resilience and prefrontal cortex (PFC) responses to negative facial expressions. This would suggest that resilient youth show either lower emotional reactivity or greater prefrontal-mediated regulation of emotional responses. As this is the first resilience study to assess face processing in general, no hypotheses were formulated regarding associations between resilience and activation during face processing in general. We also investigated whether relationships between resilience and neural responses to emotional faces differed by sex. As research on sex differences in resilience is lacking, especially in youth (see [19]), this analysis was also exploratory.

## Methods

### Dataset and ethical considerations

This study used data from a subset of participants who took part in the European multi-site FemNAT-CD study, set up to investigate sex differences in Conduct Disorder (CD; see [26]). It was approved by the European Commission, the funder, and local ethical committees at all participating sites. Neuroimaging data were collected at five sites (see Online Resource 1 for distribution of participants at each site).

### Participants

Useable fMRI data for the facial emotion processing task were available for 428 participants, and resilience scores were available for n = 215 of these. One participant was excluded for having an IQ below 70, and a further six participants were excluded due to excessive head movement > 3 mm), leaving 208 participants (98 males (47.1%) and 110 females (52.9%)) in the final analysis (see Online Resource 13 for sample selection flowchart). Of these, 51 (24.5%; 32 males and 19 females, all aged 9–18 years) youth had a diagnosis of CD and potentially other psychiatric disorders, and 157 were typically-developing (TD; 66 male and 91 female, 9–18 years). The CD participants were allowed to have comorbid disorders, whereas having a current psychiatric disorder or a past disruptive behaviour disorder (CD or oppositional defiant disorder) were exclusion criteria for the TD group. IQ was estimated using the two-subtest version of the Wechsler Abbreviated Scale of Intelligence (WASI; [27]) at the UK sites, and the Wechsler Intelligence Scale for Children (WISC-IV; [28]) at the other sites.

## Measures

### Resilience scores

Continuous resilience scores were computed using a residual regression approach, as outlined in Cornwell et al. [24]. Resilience scores were calculated for each participant based on a range of parent- and self-reported measures of adversity and symptoms of psychopathology (both internalising and externalising disorders). To assess exposure to adversity and trauma such as childhood maltreatment or neglect, a parent-reported interview (the Children’s Bad Experiences questionnaire (CBE; [29]), a self-reported questionnaire (the Childhood Experiences of Care and Abuse questionnaire (CECA-Q; [30]), and the PTSD section of the Kiddie-Schedule for Affective Disorders and Schizophrenia interview (K-SADS-PL; completed by both children and parents / caregivers; [31]) were used. These three measures resulted in 45 different adversity variables.

Current and lifetime psychopathology was assessed using the K-SADS-PL and a parent-report questionnaire assessing psychopathology in a dimensional way (the Child Behaviour Checklist (CBCL; [32]). This resulted in 130 psychopathology variables. The K-SADS-PL is a semi-structured diagnostic interview based on DSM-IV-TR criteria [33] and was used to measure psychopathology categorically and provide information regarding severity and functional impairment, whereas the CBCL measured psychopathology dimensionally. Together, these measures assessed symptoms of a broad range of internalising and externalising disorders, including depression, anxiety, and CD.

To calculate a resilience score for each individual, variables related to adversity exposure and lifetime psychopathology were entered into two separate exploratory factor analyses which served as a method of data reduction and revealed the underlying structure of the dataset. An exploratory factor analysis was used as there were no a priori hypotheses with regards to latent dimensions that might be identified by the process. As the sample size exceeded 250, all factors with an eigenvalue larger than 1 were retained [34] (for a list of factors and the cumulative % of variance explained, see Online Resource 2). Some factor scores were inverted, such that higher scores for all factors represented greater adversity exposure or higher levels of psychopathology. The resulting factor scores were weighted by variance explained and then aggregated using the median operator (separately for adversity exposure and lifetime psychopathology) to reduce the influence of outliers. We then tested for the expected positive relationship between adversity and psychopathology in a simple linear regression model and found that these aggregated variables were positively correlated (*R*^*2*^ = 0.13; see Online Resource 3). Resilience scores were then generated for each participant by regressing their aggregated adversity exposure score against their aggregated psychopathology score and then calculating their distance from the regression line along the psychopathology dimension. Individuals who were lower than expected in psychopathology, given their level of adversity exposure, had higher resilience scores, and vice-versa. Resilience scores could range from -1 to 1, with higher scores indicating higher resilience, but in practice a more restricted range was observed (-0.47 to 0.22).

### Image acquisition and pre-processing

All sites underwent site qualification procedures prior to commencing data collection to ensure comparability of the fMRI data (see [35] for details). Functional data were collected using echo-planar T2-weighted imaging (EPI) sensitive to the blood-oxygen-level-dependent (BOLD) signal contrast covering the whole brain (TE = 30 ms, TR = 2500 ms, voxel size = 3 × 3x3mm; flip angle = 83 degrees; no. of slices = 192, slice thickness = 2 mm).

Neuroimaging data were pre-processed and analysed using SPM12 (https://www.fil.ion.ucl.ac.uk/spm/software/spm12/). Prior to the main analysis, pre-processing steps and quality control procedures (e.g., visual inspection of the data at each step in the pre-processing pipeline and checking for signal dropout in the amygdala and / or PFC signal dropout) were performed. First, functional scans were interpolated to correct for slice-timing differences and realigned to the first scan by rigid body transformations to correct for head movement. Second, the realigned images were then co-registered to the T1 standard template in Montreal Neurological Institute (MNI) space. Third, a normalisation process registered images from all participants to the same coordinates to ensure compatibility between participants’ brain areas. Fourth, smoothing was applied to increase the signal-to-noise ratio. A smoothing kernel of 6 mm full-width at half-maximum was used to maintain a balance between image resolution and signal-to-noise ratio. Participants who showed head movement that exceeded the size of one voxel (i.e., > 3 mm) were excluded.

### Conscious face processing task

Participants viewed greyscale photographs of angry, fearful, and neutral faces posed by 30 different identities (50% female) taken from the NimStim Face Stimulus Set [36] and were asked to press either the left button on a button box to indicate that the face was male, or the right button to indicate that the face was female [37]. Stimuli were presented in 17.5-s epochs consisting of 5 faces from the same category (angry, fearful or neutral) intermixed with 5 null events (fixation cross). Each trial consisted of the presentation of a face for 1000 ms, followed by a fixation cross (750 ms). Null events involved a 1750 ms presentation of a fixation cross. During each epoch, the stimuli were pseudorandomised with respect to trial type (either faces or null events) and sex to enhance design efficiency while ensuring the stimulus presentation order was unpredictable [38]. Twelve epochs of each emotion category were presented (60 angry, 60 fearful, and 60 neutral faces) resulting in 180 facial expressions and 180 null events overall. Total task duration was 10-min and 30-s. For a visual depiction of the task, see Online Resource 14. Reaction time (RT) and accuracy of sex discrimination were measured during the task. Finally, we performed a one-sample t-test for the key contrasts of interest (anger > neutral/ fear > neutral) to examine the main effects of task (see Online Resource 16).

### fMRI analyses

For each participant, a general linear model was created in SPM12 to estimate parameters. First-level models consisted of the four experimental regressors (angry, fearful and neutral face trials, and fixation) and six realignment parameters accounting for motion. A high-pass filter was used to remove low-frequency signal drift (cut-off: 128 s). At the first level, contrast images were generated to assess the effect of each condition (angry > neutral; fearful > neutral; neutral > fixation) for each participant. Within the emotion contrasts, neutral expressions served as a baseline. The neutral > fixation contrast was included to examine whether there were resilience effects on neural responses to faces in general.

Second-level analyses then tested for associations between resilience scores and activation in response to each experimental condition. Resilience scores were included as a variable of interest, and group, sex, age, and site (site variables recoded from integer to binary data using ‘one-hot encoding’, see [39]) were added as covariates of no interest. One-sample t-tests were performed to assess whether mean activation at each voxel in the brain was significantly different from zero across participants. Both positive and negative effects were tested for.

Two approaches were used to threshold second-level maps. Firstly, a whole-brain analysis was performed using an activation threshold of *p* ≤ 0.001, uncorrected, and applied cluster-based FWE correction (*p* < 0.05) to clusters of significant voxels. Secondly, we conducted a region-of-interest (ROI) analysis using a *p* < 0.05, Family-Wise Error (FWE) correction for multiple comparisons in small volumes (i.e., small-volume correction) in the amygdala, hippocampus, orbitofrontal cortex, dorsolateral PFC, and inferior frontal gyrus. ROI masks were created individually for each region using the Wake Forest University (WFU) Pickatlas toolbox (Version 3.0.5; [40]). Our selection of ROIs was informed by our recent systematic review [21], and anatomical regions were defined using the AAL3 toolbox for SPM12 [41]. A sex-by-resilience interaction term was created to test for interactions between sex and resilience on brain activity. Finally, we repeated the analyses in the TD and CD groups separately, to exclude the possibility that our findings were driven by group effects (as the TD and CD groups significantly differed in resilience scores, see Online Resource 4) Table 1.

**Table 1:** Demographic and clinical characteristics of the sample (*N* = 208)

Variable	*M*	*SD*	Range
Age (years)	13.28	2.61	9–18
Estimated IQ	103.64	11.84	73–138
Resilience Score	0.01	0.11	– 0.47 to 0.22

	*N*	%	M *(SD)* Symptoms Across Full Sample
Sex (M/F)	98 / 110	47.1 / 52.9	–
CD Diagnoses	51	24.5	1.23 (2.29)
ADHD Diagnoses*	28	13.5	1.19 (2.61)
MDD Diagnoses*	12	5.8	0.61 (1.74)
GAD Diagnoses*	8	3.8	0.17 (0.78)

Note: Diagnoses of Conduct Disorder (CD) and comorbid disorders were made using the Kiddie-Schedule for Affective Disorders and Schizophrenia-Present and Lifetime version. *ADHD* attention-deficit/hyperactivity disorder, *MDD* major depressive disorder, *GAD* generalised anxiety disorder. *These individuals represent subsets of the above CD group because typically developing youth with other disorders were excluded at the screening stage

For future meta-analyses and the resilience field as a whole, regions present at a threshold of *p* ≤ 0.001 with a cluster size (*k*) of ≥ 10 voxels are reported in Table 2, which is a more stringent threshold than that recommended by Lieberman and Cunningham [42]. However, only those findings that survive FWE correction will be the focus of the Results and Discussion sections.

**Table 2:** Brain regions that were significantly associated with resilience in the overall sample, together with their cluster sizes and coordinates

	Region	Hemisphere	T-value	Cluster Size	MNI Coordinates		
					X	Y	Z
**Fear > Neutral**							
*Positively Correlated*	Inferior Frontal Gyrus*	**Left**	**5.07**	**36**	**– 51**	**18**	**3**
	Precentral Gyrus	Left	4.05	12	**– **39	6	39
	**Inferior Frontal Gyrus**	**Right**	**3.85**	**49**	**48**	**27**	**-9**
	Paracentral Lobule	Right	3.57	40	0	**– **42	63
	Middle Temporal Gyrus	Left	3.56	25	**– **54	**– **39	**– **3
	Inferior Frontal Gyrus / operculum	Right	3.51	16	51	15	18
*Negatively Correlated* **Anger > Neutral**	Lobule IV, V of cerebellum	Left	3.27	12	**– **12	**– **33	**– **12
*Positively Correlated*	Precentral Gyrus	Left	3.97	33	**– **39	6	39
**Neutral > Fixation**	Middle Temporal Gyrus	Right	3.72	11	48	**– **27	**– **6
*Negatively Correlated*	Superior Temporal Gyrus*	**Right**	**5.15**	**91**	**51**	**– 27**	**– 3**
	Supplementary Motor Area	Left	4.80	13	**– **6	3	63
	Middle Temporal Gyrus	Right	4.52	77	51	**– **54	9
	Middle Temporal Gyrus	Left	4.16	18	**– **51	**– **39	**– **3
	Inferior Occipital Gyrus	Right	3.93	12	39	**– **81	**– **6
	Fusiform Gyrus	Right	3.83	38	33	**– **48	**– **18
	Putamen	Left	3.77	14	**– **21	9	3
	**Hippocampus**	**Left**	**3.76**	**26**	**– 27**	**– 6**	**– 21**
	**Inferior Frontal Gyrus**	**Right**	**3.73**	**33**	**54**	**24**	**12**
	Middle Frontal Gyrus	Right	3.58	13	42	39	6
	Precentral Gyrus	Right	3.53	14	27	**– **24	51
	Middle Cingulate Gyrus	Right	3.51	26	9	21	30
	Middle Temporal	Right	3.49	14	45	**– **69	**– **3
	Caudate	Right	3.45	18	18	9	15

Note: All regions significant at *p* ≤ .001, uncorrected, and k ≥ 10. Results shown in bold denote that the area survived family-wise error (FWE) small-volume correction (*p* < .05). * *p* < .05, FWE whole-brain correction

## Results

### Demographic and clinical characteristics

Demographic and clinical characteristics of the sample are reported in Table 1. Resilience scores ranged from  – 0.29 to 0.22 in the TD group, and from  – 0.47 to 0.17 in the CD group (where higher scores indicate greater resilience). The CD participants had lower resilience scores on average (M_resilience_ =  – 0.04) than the TD participants (M_resilience_ = 0.03; F = 13.98, *p* < 0.001; for group and sex differences in terms of age, estimated IQ, psychopathology symptoms, and interaction effects, see Online Resource 4).

### Behavioural data

As the data were not normally distributed (see Online Resource 5), we used Spearman’s rho to test for correlations between resilience scores and accuracy of sex discrimination and reaction time (RT) for correct responses (see Online Resource 6). Due to a task coding issue (only responses made within 750 ms of the stimulus onset were measured), data were missing for 9 participants (4.3%). No significant associations were observed between resilience scores and sex discrimination accuracy or RTs for correct responses.

## fMRI results

### Main effects of task

We observed main effects of emotion in the right middle temporal gyrus for the fear contrast and in the right pars triangularis for the anger contrast (both significant at *p* < 0.001, FWE whole-brain corrected; see Online Resource 16).

### Correlations between resilience scores and neural responses to fear > neutral

**Fig. 1:**
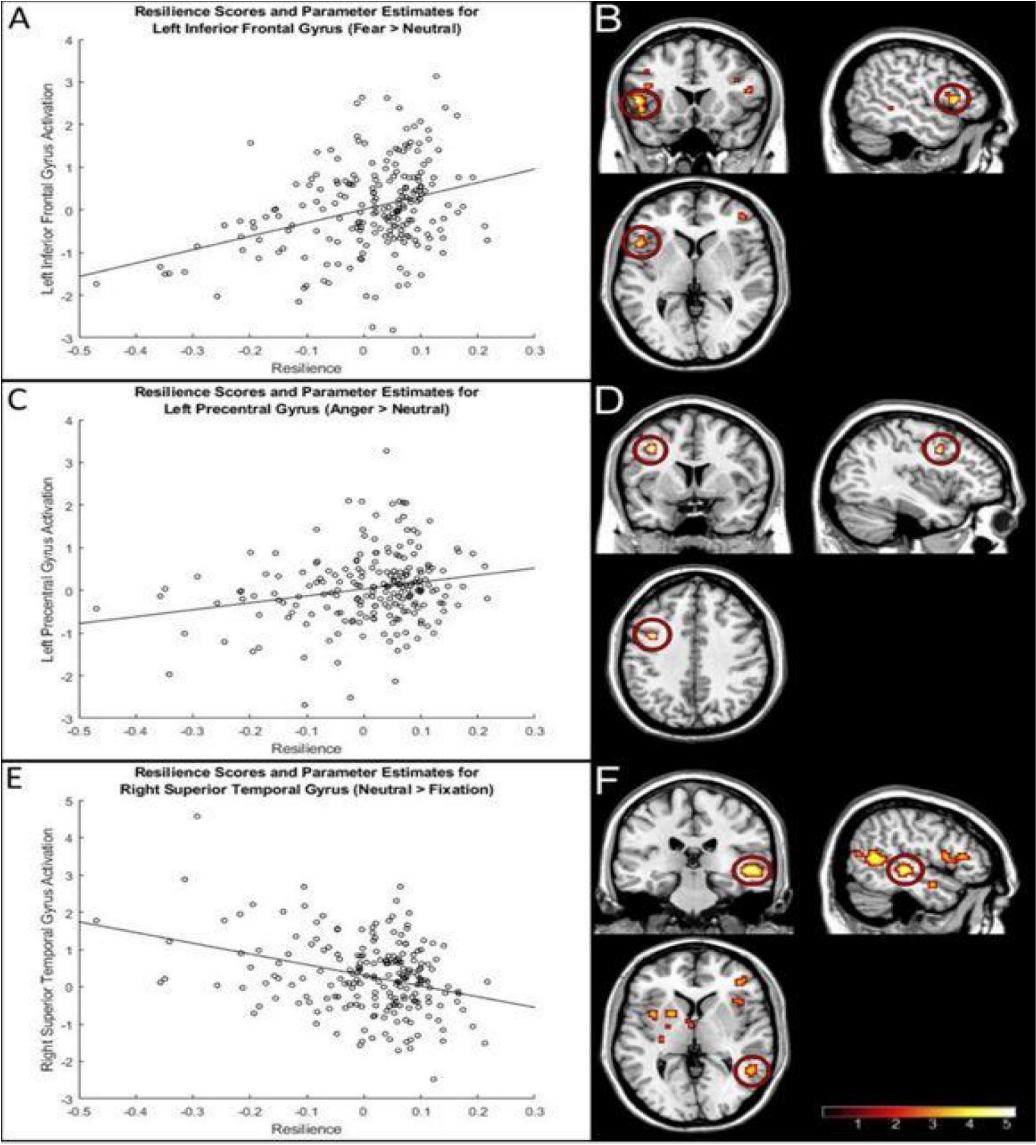
Associations between resilience and brain activation to emotional and neutral faces in the overall sample. Panels A, C, and E show scatterplots of correlations between resilience scores and brain activation. Panels B, D, and F show statistical parametric maps of the region of peak activation for the corresponding contrast. The region shown in each scatterplot is highlighted in the brain maps using red ellipses, and the colour bar represents t-values. Maps are presented at *p* =.001, uncorrected

We observed positive associations between resilience and activation in regions of the PFC for the fear > neutral contrast, most notably the left (*t* = 5.07, *p* < 0.05, FEW-whole-brain-corrected; see Fig. 1 and Table 2) and right (*t* = 3.85, *p* < 0.01, FWE-SVC) inferior frontal gyrus. No significant relationships were found between resilience and activation in the other ROIs (e.g., amygdala, hippocampus).

### Correlations between resilience scores and neural responses to anger > neutral

No associations survived whole-brain FWE correction for this contrast and no significant associations were observed in the ROIs.

### Correlations between resilience scores and neural responses to neutral > fixation

No positive associations between resilience and brain activation were found for the neutral > fixation contrast. However, the ROI analyses revealed negative associations between resilience and brain activation in the right superior temporal gyrus (*t* = 5.15, *p* < 0.05, FWE-whole-brain-corrected; see Fig. 1 and Table 2), right inferior frontal gyrus (*t* = 3.73, *p* < 0.05, FWE-SVC), and left hippocampus (*t* = 3.76, *p* < 0.05, FWE-SVC). Resilience was not associated with activation in the other ROIs.

### Sex-by-resilience score interactions

Although sex-by-resilience interactions were observed in several regions (see Online Resource 7 for details), none of these associations survived whole-brain FWE correction or were present at *p* < 0.05, FWE-SVC in the regions of interest.

### Sensitivity analyses

As the TD and CD groups differed in resilience scores, we repeated the analysis in each group separately to ensure that the resilience effects reported above were not explained by group differences in brain activation. Findings for the TD group (n = 157) were similar to the main findings (resilience was positively associated with bilateral inferior frontal gyrus activation to fearful faces and negatively associated with right inferior frontal gyrus activation to neutral faces; see Online Resource 8), with the exception of the negative association between resilience and right superior temporal gyrus activation for neutral faces which was rendered non-significant. However, none of the results for the CD group (n = 51) survived FWE whole-brain correction or FWE-SVC correction in the ROIs (see Online Resource 9). Analyses investigating males and females separately also yielded similar findings to those reported in the full sample, though the findings for males only were stronger than those for females only (i.e., higher *t* values and FWE whole-brain corrected findings; see Online Resources 10–11).

The age range of our sample was relatively wide (9–18 years). Due to the changes in brain function that can occur during development, we repeated the analysis using the subset of the sample (*n* = 153) that had started pubertal development (i.e., those who scored 3 or above on the Pubertal Development Scale; [43]). We focused on pubertal development, rather than chronological age, to ensure that the sample was relatively homogenous in terms of development, and because puberty is a time when many people develop psychiatric disorders [44], pre-pubertal participants may not have reached the stage of increased risk for developing disorders, especially depression. Although this analysis revealed findings for the fear > neutral contrast that were similar to the main analysis (left inferior frontal gyrus activation was positively associated with resilience; see Online Resource 12), none of these associations survived FWE whole-brain or small-volume correction.

Finally, we reran the analysis excluding ‘Group’ as a nuisance variable to ensure that the inclusion of this variable did not drastically alter the results. We found similar results, with the left and right IFG showing positive associations with resilience for the fear > neutral contrast, and the right superior temporal gyrus showing negative associations with resilience for the neutral > fixation contrast.

## Discussion

Using a novel data-driven and dimensional definition of resilience, we investigated associations between resilience and neural responses to negatively-valenced facial expressions (i.e., angry or fearful faces), and faces in general, in an adolescent sample. Resilience was positively associated with left inferior frontal gyrus activation during fear processing, with a similar (albeit weaker) association observed in the right inferior frontal gyrus. No positive associations were observed between resilience and brain activation when processing neutral faces (neutral > fixation contrast). Instead, we found that resilience was negatively associated with right superior temporal gyrus, left hippocampus, and right inferior frontal gyrus responses to neutral faces.

Contrary to our first hypothesis, resilience was not associated with lower amygdala responses to fearful or angry faces. However, consistent with predictions, we found that resilience was positively correlated with prefrontal cortex responses to fearful facial expressions, particularly in the inferior frontal gyrus. This suggests enhanced regulatory control of emotional responses in youth higher in resilience, compared to their more vulnerable counterparts.

It should be noted that previous studies examining resilience and facial emotion processing in adults have operationalised resilience in various ways. Nevertheless, the left inferior frontal gyrus is implicated in emotion regulation [45], which has been proposed elsewhere as an important mechanism of resilience [46–48]. More broadly, the inferior frontal gyrus has been implicated in cognitive control, which is the ability to attend to task-relevant stimuli and ignore irrelevant stimuli [49, 50]. In other words, cognitive control is necessary to override automatic behaviours and select a response appropriate to a given situation. Previous neuropsychological [51, 52] and neuroimaging [53] studies have proposed enhanced cognitive control as a mechanism that underlies resilient functioning. Correspondingly, a review by McTeague et al. [54] found *deficits* in cognitive control across psychiatric disorders, suggesting they are a transdiagnostic risk factor for psychopathology. Additionally, a review by McRae et al. [55] suggested that cognitive control may be an important factor in reappraisal (a facet of emotion regulation), which has been linked to resilience in other studies (e.g., [14]).

Previous studies from our lab have examined grey matter volume [24] and cortical structure [25] as markers of resilience to adversity in an overlapping youth sample, using the residual regression approach to determining resilience. The first study found that resilience was positively correlated with grey matter volume in the right inferior frontal gyrus, which is consistent with our finding that right inferior frontal gyrus activation was positively associated with resilience. However, other regions identified as associated with resilience in these studies (e.g., right medial frontal gyrus, superior frontal gyrus and lateral occipital gyrus) were not observed in our fMRI analysis. The similarity between findings from the present study and those from the voxel-based morphometry (VBM) study may be because VBM and fMRI are similar in terms of their methodology (i.e., both use voxel-based analysis to investigate differences across the entire brain, rather than distinguishing between cortical and subcortical regions). Nevertheless, these findings collectively indicate the importance of the inferior frontal gyrus in resilient functioning in youth.

Together with these previous findings, and those from the wider literature, our finding of a positive association between resilience and bilateral inferior frontal gyrus activation when viewing fearful faces suggests that resilient youth may possess enhanced cognitive control abilities. This may enable them to regulate their emotional responses to fearful facial expressions more effectively than those lower in resilience.

No positive associations between resilience and brain activation were observed when examining brain activation during face processing in general. However, resilience was negatively correlated with activation in the right superior temporal gyrus for this contrast. To our knowledge, this area has not been identified in previous neuroimaging studies of resilience but is highly connected to the PFC and the limbic system and has been implicated in social cognition [56]. In addition, we observed a negative association between resilience and left hippocampus activation for this contrast. The hippocampus forms part of the limbic system and plays a key role in memory and emotion processing [57]. Taken together, these findings suggest that less resilient individuals could experience heightened affective responses to neutral faces, potentially due to difficulties in interpreting neutral faces and perceiving them as threatening [58]. We also observed a *negative* association between resilience and right inferior frontal gyrus activation during face processing in general (i.e., indexed by the contrast of neutral faces vs fixation). The right inferior frontal gyrus is implicated in several functions including response inhibition and emotion processing [59], and this finding supports a previous, longitudinal study that examined resilience and face processing in young people at risk for a mood disorder [60].

However, we found the same area to be positively associated with resilience when processing fearful faces. Furthermore, as mentioned previously, an earlier study from our lab [24] examined structural correlates of resilience in young people and found an *increase* in right inferior gyrus volume in resilient youth. This contrast in associations between resilience and brain activation patterns during fearful vs neutral vs neutral face processing is intriguing and suggests that the right inferior frontal gyrus plays a key, but as yet not entirely clear, role in resilient functioning. It should be noted that our analysis of associations between resilience and brain activation during neutral face processing was done in an exploratory fashion, so we recommend that future fMRI studies of resilience investigate brain responses to neutral (as well as emotional) faces by including a low-level baseline (e.g., fixation cross) to build on these preliminary findings.

Finally, we examined whether relationships between resilience and brain activation differed by sex. None of the observed interactions survived FWE whole-brain or small-volume correction. However, we report the uncorrected findings here for the benefit of meta-analyses and to encourage researchers to consider examining sex differences in future neuroimaging studies, building upon these preliminary results. The most interesting finding from this analysis was that resilience appeared related to right medial prefrontal activity in contrasting ways in males vs. females. However, these findings need to be followed up in larger, more representative samples. Differences in resilience and the direction of brain activation between males and females could be associated with features such as adversity type, as adolescent females are more likely to have suffered sexual abuse [61], whereas adolescent males are more likely to have suffered physical abuse [62]. Furthermore, internalising disorders such as anxiety and depression are more common in adolescent females, and externalising disorders are more prevalent in adolescent males [63]. Being low in internalising symptoms despite adversity exposure may therefore represent stronger evidence of resilience in females, and vice-versa for externalising symptoms in males. To date, very few fMRI studies have tested for potential interactions between sex and resilience [21], partly because they have not used dimensional measures of resilience. Overall, our results are consistent with Fallon et al.’s [19] review and provide tentative support for the notion that resilience mechanisms may differ by sex. Hence, future fMRI studies of resilience should test for sex*resilience interactions when investigating the relationship between resilience and brain activation, as it cannot be assumed that resilience has similar effects in males and females.

### Strengths and limitations

Our study had several strengths. Firstly, we used a data-driven, dimensional approach to defining resilience in young people which captured symptoms of many different psychiatric disorders and assessed multiple types of adversity or trauma (rather than a specific disorder or type of adversity, e.g., bullying). We also conducted two novel analyses. The first examined associations between resilience and responses to neutral faces (thus investigating whether resilience is associated with altered brain responses to faces in general), and the second tested for sex-by-resilience interactions to investigate whether associations between resilience and neural responses differed by sex.

A limitation of the current study was that it used data from a larger project designed to investigate sex differences in Conduct Disorder (CD). Although we measured and included those with additional psychiatric disorders, CD was the most prominent psychopathology factor in the residual regression analysis that was used to generate resilience scores. However, we acknowledge that resilience to other disorders may have distinct neural correlates that were not captured as well in our study. Future research should therefore aim to use population-based samples so that the samples are not comprised of a mix of individuals with full psychiatric disorders and ‘super-well’ controls.

A second limitation was the cross-sectional design. It is possible that some of those who experienced high levels of adversity but who currently show low levels of psychopathology symptoms (i.e., presenting as resilient) will go on to develop mental illness in the future. In addition, the cross-sectional design does not allow us to infer causal relationships between resilience and differences in brain activation—only associations between these variables.

A third limitation relates to our conceptualisation of resilience. Although focusing on psychopathology in the face of adversity exposure is a widely used approach in neuroimaging studies of resilience [21], a more comprehensive and valid measure of resilience would be one that considers functioning across life domains (e.g., social, academic, occupational; [64]) rather than just levels of psychopathology. Future studies should therefore aim to use broader dimensional measures of resilience and adopt longitudinal designs that would be better placed to capture the development of resilience over time. This would allow researchers to distinguish between pre-existing differences in responses to emotional stimuli that confer resilience in the face of adversity (i.e., akin to trait resilience) and positive adaptations in brain function that may occur following adversity exposure (i.e., which fits better with current conceptualisations of resilience as an outcome or process). Future studies could also include positively-valenced facial expressions to investigate whether resilience is associated with brain activation during the processing of positive stimuli (e.g., heightened responses in reward-related regions). It would also be of interest to investigate emotion regulation more directly, for example using a cognitive reappraisal task, to examine whether resilience is associated with heightened activation in brain regions involved in emotion regulation. Lastly, our measure of resilience was study-specific – it was based on the combination of the psychopathology and adversity measures available in our sample, and the relationship between these variables in this sample. Future progress in the field will require the development of continuous resilience measures that can be derived and applied across studies.

## Conclusion

Consistent with our hypothesis that resilience would be positively associated with activity in brain regions implicated in emotion regulation, resilient individuals showed increased activation in prefrontal areas when processing fearful facial expressions.. We also observed negative correlations between resilience and activity in the superior temporal gyrus and other limbic regions (e.g., hippocampus) during the processing of neutral faces. Resilient youth showed lower neural responses to neutral facial expressions in frontal, temporal and limbic regions. Although we did not measure how the expressions were perceived or appraised, the neutral stimuli may have been perceived as more threatening by those who were lower in resilience. Clinically, understanding that those who are less resilient are more likely to have heightened reactivity to neutral faces (i.e., perhaps because they misinterpret them as angry or fearful), could have implications for future interventions designed to enhance resilience. Our hypothesis that resilience would be negatively associated with amygdala responses to negatively-valenced stimuli (either fearful or angry faces) was not supported. In addition, although our sex-by-resilience interaction analyses revealed distinct associations between resilience and activity in some areas in males versus females (e.g., positive associations with medial prefrontal cortex activity in males, but negative associations in females), these associations did not survive correction for multiple comparisons. Nevertheless, these findings may have implications for future neuroimaging research on resilience using mixed-sex samples, as they provide preliminary evidence that associations between resilience and neural responses to emotional stimuli may differ between the sexes.

## Supplementary Information

Below is the link to the electronic supplementary material.

Supplementary file1 (PDF 516 KB)

## Data Availability

No datasets were generated or analysed during the current study.
