# Supplementary material for: Neural activation during processing of emotional faces as a function of resilience in adolescents
Source: Eur Child Adolesc Psychiatry. 2025 Apr 10;34(9):2943–55. doi: 10.1007/s00787-025-02703-y (PMC12507940; doi:10.1007/s00787-025-02703-y)
Supplement: Supplementary file 1 — Supplementary file1 (PDF 516 KB) [file 787_2025_2703_MOESM1_ESM.pdf]

Neural activation during processing of emotional faces as a function of resilience in adolescents

European Child & Adolescent Psychiatry

Steve Eaton[1], Harriet Cornwell[1], Jack Rogers[2], Stephane De Brito[2], Nicola Toschi[3], Christina Stadler[4], Nora Raschle[4], Kerstin Konrad[5], Gregor Kohls[5,6], Areti Smaragdi[7], Karen Gonzalez-Madruga[8], Maaike Oosterling[1], Anne Martinelli[9,10], Anka Bernhard[9], Christine M Freitag[9], Catherine Hamilton-Giachritsis[1], and Graeme Fairchild[1]

Authors' affiliations:

1 Department of Psychology, University of Bath, Bath, UK

2 School of Psychology, University of Birmingham, Birmingham, UK

3 Department of Biomedicine and Prevention, University of Rome 'Tor Vergata', Rome, Italy

4 Jacobs Center for Productive Youth Development at the University of Zurich, Zurich, Switzerland

5 Child Neuropsychology Section, Department of Child and Adolescent Psychiatry, Psychosomatics, and Psychotherapy, University Hospital RWTH, Aachen, Germany

6 Department of Child and Adolescent Psychiatry, Faculty of Medicine, TU Dresden, Dresden, Germany

7 Child Development Institute, Toronto, Canada

8 Department of Child and Adolescent Psychiatry, King's College London, London, UK

9 Department of Child and Adolescent Psychiatry, Psychosomatics, and Psychotherapy, University Hospital Frankfurt, Goethe University Frankfurt, Frankfurt am Main, Germany

10 School of Psychology, Fresenius University of Applied Sciences, Frankfurt am Main, Germany

Corresponding author: Steve Eaton, Department of Psychology, 10 West, University of Bath, Bath, BA2 7AY, United Kingdom. ORCiD No: 0000-0002-0021-2391

Email: eatons@cardiff.ac.uk

Supplementary Materials

Online Resource 1, Number of Participants from each Site (N=208)

Site Number	Site Name	*n*
1	Frankfurt (DE)	53
2	Aachen (DE)	65
4	Southampton (UK)	22
5	Basel (CH)	0
7	Birmingham (UK)	68

Online Resource 2, Adversity and Psychopathology Factors, and Cumulative % of Variance Explained, for Deriving Resilience Scores

Adversity Factor	Cumulative % of Variance Explained	Psychopathology	Cumulative % of Variance Explained
Emotional support from Father figure	22.71	Current symptoms of externalising disorder	16.57
Neglect	30.78	Past Oppositional Defiant Disorder symptoms	21.43
Relationship with Mother figure	37.43	Past Depressive Disorder symptoms	25.62
Physical abuse	43.17	Substance Use	29.44
Interest / Concern from Father figure about social relationships	47.44	Lifetime Bulimia Nervosa symptoms	32.08
Emotional support from Mother figure	51.21	Current self-harm and suicidality	34.49
Sexual Abuse	54.36	Lifetime autistic spectrum disorder symptoms	36.67
Exposed to a violent crime	57.34	Current Separation Anxiety Disorder issues	38.67
Lack of parental supervision	59.96	Lifetime avoidant disorder / social phobia	40.57
Emotional abuse from parents	62.42	Past agoraphobia symptoms	42.33
Other trauma	64.65	Lifetime racing thoughts	43.94
		Lifetime Obsessive Compulsive Disorder symptoms	45.5
		Substance-related disorders	46.99
		Past use of weight loss methods	48.45
		Lifetime tic disorder symptoms	49.87
		Lifetime mania symptoms	51.25
		Current use of weight loss methods	52.6
		Past self-harm and suicidal ideation	53.91
		Past ADHD symptoms	55.13
		Current eating binges / attacks	56.33

Lifetime stereotyped or repetitive behaviours	57.52
CBCL Subscales	58.66
Lifetime school reluctance / refusal	59.76
Lifetime delusions	60.81
Past separation anxiety issues	61.83
Lifetime use of exercise for weight loss	62.81
Lifetime emaciation	63.78
Current agoraphobia symptoms	64.74
Lifetime somatic complaints	65.66
Lifetime repetitive behaviour	66.58
Past suicidality	67.47
Current irritiability	68.33
Current depressive / panic disorder symptoms	69.15
Past conduct problems	69.95
Lifetime social communication deficits	70.74
Lifetime worry / fear	71.51

Online Resource 3, Scatterplot illustrating the association between adversity exposure and psychopathology symptoms (from Cornwell et al., 2023).

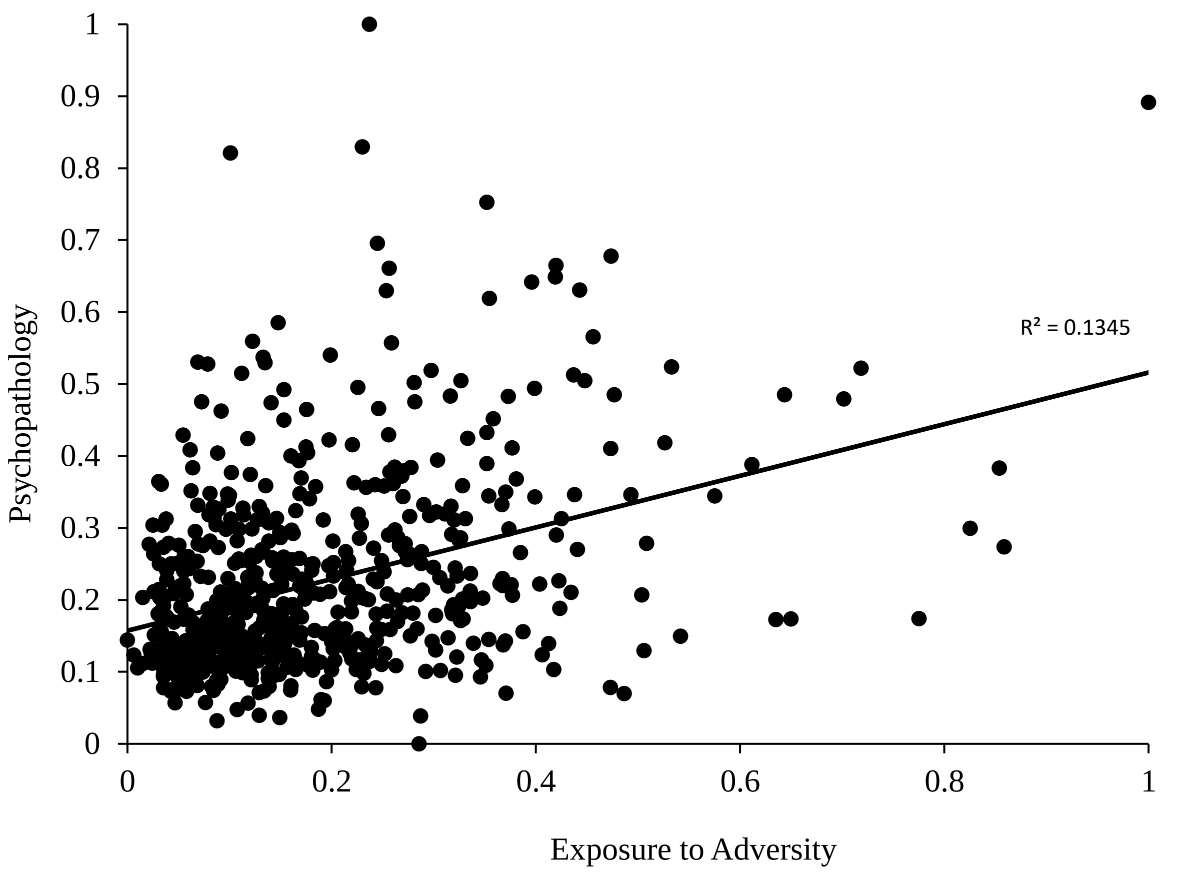

Online Resource ~~3~~4, Demographic and clinical characteristics of the sample, separated by group and sex

Characteristic	Male TD (*n*=66)		Male CD (*n*=32)		Female TD (*n*=91)		Female CD (*n*=19)		Group (CD/TD) Effects		Sex (Male / Female) Effects		Sex-by-Group interaction	
	M	SD	M	SD	M	SD	M	SD	F	*p*	F	*p*	F	*p*
Age	12.94	2.54	12.56	2.15	13.6	2.83	14.16	2.12	0.04	0.85	6.46	0.01	13.8	0.24
Estimated IQ	105.8	12.43	96.62	12.14	105.2	10.06	100.6	13.12	13.17	<.001	0.63	0.43	1.73	0.19
Resilience score	0.04	0.08	-0.04	0.16	0.02	0.09	-0.04	0.15	13.91	<.001	0.22	0.64	0.26	0.61
CD symptoms	0.14	0.43	4.31	1.67	0.13	0.43	4.16	2.29	509.38	<.001	2.16	0.14	1.45	0.23
ADHD Symptoms	0.09	0.54	5.53	3.02	0.03	0.24	3.42	3.39	294.52	<.001	16.56	0.001	16.64	<.001
MDD Symptoms	0.12	0.66	1.53	2.50	0.09	0.66	3.26	3.03	91.09	<.001	12.50	0.001	13.50	<.001
GAD Symptoms	0.07	0.40	0.47	1.19	0.04	0.33	0.58	1.74	13.41	<.001	0.10	0.76	0.31	0.58

Note: TD = Typically-Developing; CD = Conduct Disorder / other comorbid disorders

Online Resource ~~4~~5, Normality tests for behavioural data

	Shapiro-Wilk*	Skewness	Kurtosis

Resilience Scores	0.90	-1.40	2.57
Fear Accuracy	0.94	-0.73	3.18
Fear RT	0.82	-0.29	9.99
Angry Accuracy	0.96	-0.53	-0.46
Anger RT	0.84	1.69	3.18
Neutral Accuracy	0.96	-0.54	-0.26
Neutral RT	0.79	-2.60	25.41

**All Shapiro-Wilk values significant at p=<.001*

Online Resource ~~5~~6, Correlations between resilience, task accuracy and reaction time (using Spearman's Rho)

	Resilience Scores	
	ρ	*p*
Fear Accuracy	-.11	0.14
Fear RT	0.03	0.69
Anger Accuracy	-.04	0.58
Anger RT	0.001	0.99
Neutral Accuracy	-.07	0.33
Neutral RT	0.04	0.58

Online Resource ~~6~~7 – Brain regions, clusters size and coordinates that were significantly associated with the resilience-by-sex interaction term

	Region	Hemisphere	T-value	Cluster Size	MNI Coordinates		
					X	Y	Z
**Fear > Neutral**							
Negatively Correlated	Fusiform Gyrus	Left	4.46	38	-33	-66	-12
	Superior Frontal Gyrus, Medial	Right	4.07	43	12	48	24
	Fusiform Gyrus	Left	3.97	28	-36	-3	-27
	Inferior Frontal Gyrus	Right	3.47	35	39	30	12
	Lobules IV, V of Cerebellum	Right	3.47	36	12	-54	-18
	Middle Temporal Gyrus	Right	3.41	16	60	-54	18
	Fusiform Gyrus	Right	3.38	25	36	-39	-15
	Inferior Temporal Gyrus	Left	3.31	13	-45	-42	-12
	Precuneus	Left	3.30	12	-15	-48	18
	Middle Frontal Gyrus	Right	3.10	19	39	30	33
	Fusiform Gyrus	Right	3.05	23	21	-39	-15
	Superior Temporal Gyrus	Left	2.96	11	-51	-42	12
**Anger > Neutral**							
Negatively Correlated	Middle Occipital Gyrus	Right	4.16	41	54	-66	24
	Temporal Pole: Superior Temporal Gyrus	Right	3.80	19	48	6	-18
	Superior Frontal Gyrus	Right	3.24	10	21	30	39
	Posterior Cingulate Gyrus	Left	3.23	19	-6	-36	12
	Superior Frontal Gyrus, Medial	Right	3.19	10	9	51	30
	Crus 1 of Cerebellum	Left	3.00	12	-15	-81	-21
**Neutral > Fixation**							
Positively Correlated	Precentral Gyrus	Right	3.81	22	42	0	30
	Inferior Temporal Gyrus	Right	3.81	11	57	-45	-12
	Supplementary Motor Area	Right	3.62	13	12	15	57
	Hippocampus	Left	3.19	11	-30	-30	-12
Negatively Correlated	Angular Gyrus	Left	2.99	10	-36	-54	36

Note: All regions significant at $p \leq .001$, uncorrected, and cluster size (k) ≥ 10

Online Resource ~~7~~8 – Brain regions, cluster size and coordinates significantly associated with resilience – in typically-developing adolescents considered alone (n=157)

	Region	Hemisphere	T-value	Cluster Size	MNI Coordinates		
					X	Y	Z
**Fear > Neutral**							
Positively Correlated	Middle Frontal Gyrus	Right	3.64	19	36	36	12
	Inferior Frontal Gyrus	Right	3.61	55	48	27	-9
	Inferior Frontal Gyrus	Left	3.49	22	-45	18	-6
	**Inferior Frontal Gyrus**	**Left**	**3.47**	**11**	**-57**	**12**	**21**
	Inferior Temporal Gyrus	Right	3.45	23	48	-57	-21
	**Inferior Frontal Gyrus**	**Right**	**3.43**	**21**	**48**	**18**	**12**
	Middle Cingulate Gyrus	Right	3.40	14	21	-48	36
Negatively Correlated	Fusiform Gyrus	Right	3.62	19	36	-42	-12
	Lobules IV, V of cerebellum	Left	3.57	19	-9	-33	-12
	Lobule III of cerebellum	Right	3.42	18	12	-33	-12
**Anger > Neutral**							
Positively Correlated	Caudate	Right	4.36	19	18	18	12
	Middle Temporal Gyrus	Right	3.98	22	48	-27	-6
	Inferior Frontal Gyrus	Left	3.53	19	-33	27	-6
**Neutral > Fixation**							
Negatively Correlated	Fusiform Gyrus	Right	4.70	88	42	-48	-15
	Middle Temporal Gyrus	Right	4.39	43	54	-30	-3
	Mediodorsal medial magnocellular	Right	4.26	24	6	-18	6
	Inferior Occipital Gyrus	Right	4.23	31	42	-81	-9
	Middle Temporal Gyrus	Right	4.20	66	54	-48	6
	Rolandic Operculum	Left	4.19	18	-39	-27	21
	Insula	Left	4.09	25	-42	3	3
	Putamen	Right	4.05	12	36	0	-9
	**Inferior Frontal Gyrus**	**Right**	**4.00**	**15**	**45**	**18**	**9**
	Lingual Gyrus	Right	3.85	11	12	-87	-9

Note: All regions significant at $p \leq .001$, uncorrected, $k \geq 10$. Results shown in bold denote that the area survived family-wise error (FWE) whole-brain correction ($p < .05$)

Online Resource ~~8~~9 – Brain regions, cluster size and coordinates significantly associated with resilience in the Conduct Disorder group only (n=51)

	Region	Hemisphere	T-value	Cluster Size	MNI Coordinates		
					X	Y	Z
**Fear > Neutral**							
Positively Correlated	Inferior Frontal Gyrus	Left	4.72	18	-51	18	6
	Rolandic Operculum	Right	4.25	12	42	6	18
**Anger > Neutral**							
Positively Correlated	Superior Temporal Gyrus, Temporal Pole	Left	3.93	10	-42	15	-24
	Precentral Gyrus	Right	3.30	11	45	3	27
**Neutral > Fixation**							
Positively Correlated	Angular Gyrus	Right	3.58	18	39	-57	51
Negatively Correlated	Supplementary Motor Area	Left	4.42	42	-3	3	66
	Cuneus	Right	4.02	25	9	-87	15
	Middle Temporal Gyrus	Right	3.65	12	45	-21	-6
	Middle Frontal Gyrus	Left	3.63	11	-27	39	18
	Amygdala	Left	3.48	10	-27	0	-21

Note: All regions significant at $p \leq .001$, uncorrected, $k \geq 10$.

Online Resource ~~9~~10 – Brain regions, cluster size and coordinates significantly associated with resilience – in males only (n=98)

					MNI Coordinates		
	Region	Hemisphere	T-value	Cluster Size	X	Y	Z
**Fear > Neutral**							
Positively Correlated	Middle Temporal Gyrus	Left	4.09	16	-51	-27	-12
	**Inferior Frontal Gyrus**	**Left**	**4.02**	**32**	**-54**	**15**	**6**
	Inferior Temporal Gyrus	Left	3.99	11	-48	-6	-27
	Hippocampus	Right	3.86	10	45	-33	-9
	Fusiform Gyrus	Left	3.63	18	-33	-66	-12
	Cuneus	Left	3.45	11	-21	-51	18
**Anger > Neutral**							
Positively Correlated	Precentral Gyrus	Left	3.82	20	-39	6	39
	Temporal Pole: Superior Temporal Gyrus	Left	3.74	13	-42	15	-24
	Middle Temporal Gyrus	Left	3.64	18	-45	-3	-27
	Posterior Cingulate	Left	3.52	18	-9	-39	12
	Supramarginal Gyrus	Right	3.50	13	63	-45	33
**Neutral > Fixation**							
Negatively Correlated	**Supplementary Motor Area**	**Left**	**5.32**	**44**	**-3**	**3**	**66**
	Superior Temporal Gyrus	Right	4.58	44	45	-24	-6
	Lobule VI of cerebellum	Right	4.58	14	27	-81	-18
	Inferior Frontal Gyrus	Right	3.88	24	36	30	-12
	Middle Frontal Gyrus	Right	3.81	15	36	-3	57
	Parahippocampal Gyrus	Right	3.80	10	27	-18	-21
	Middle Cingulate Gyrus	Right	3.75	20	9	18	33
	Precentral Gyrus	Right	3.63	13	30	-21	51

Note: All regions significant at $p \leq .001$, uncorrected, $k \geq 10$. Results shown in bold denote that the area survived family-wise error (FWE) whole-brain correction ($p < .05$)

Online Resource 11~~0~~ – Brain regions, cluster size and coordinates significantly associated with resilience – in females only (n=110)

					MNI Coordinates		
	Region	Hemisphere	T-value	Cluster Size	X	Y	Z
**Fear > Neutral**							
Positively Correlated	Inferior Frontal Gyrus	Left	3.36	14	-39	18	33
	Inferior Frontal Gyrus	Left	3.28	25	-48	18	-6
	Inferior Parietal Gyrus	Left	3.19	20	-30	-45	36
	Precuneus	Left	3.16	19	-3	-48	63
Negatively Correlated	Lingual Gyrus	Right	3.69	22	15	-33	-9
	Lobules IV, V of cerebellum	Left	3.57	18	-6	-60	-12
	Fusiform Gyrus	Right	3.52	16	36	-42	-12
**Anger > Neutral**							
Positively Correlated	Middle Temporal Gyrus	Right	3.30	12	42	-54	3
	Inferior Frontal Gyrus	Left	3.24	12	-33	42	0
	Inferior Frontal Gyrus	Left	3.15	10	-48	18	-3
**Neutral > Fixation**							
Negatively Correlated	Superior Temporal Gyrus	Right	4.25	78	42	-39	12
	Fusiform Gyrus	Right	4.02	60	42	-45	-15
	Insula	Left	3.95	21	-42	6	3
	Superior Temporal Gyrus	Right	3.68	16	48	-27	-3
	Middle Occipital Gyrus	Right	3.47	12	39	-69	0

Note: All regions significant at $p \leq .001$, uncorrected, $k \geq 10$

Online Resource 121 – Brain regions, cluster size and coordinates significantly associated with resilience in a subset of the sample with Pubertal Development scores >3 (n=153)

					MNI Coordinates		
	Region	Hemisphere	T-value	Cluster Size	X	Y	Z
**Fear > Neutral**							
Positively Correlated	Inferior Frontal Gyrus	Left	3.93	22	-51	18	3
**Neutral > Fixation**							
Negatively Correlated	Caudate	Right	4.16	11	15	0	21
	Postcentral Gyrus	Right	3.98	19	24	-33	45

Note: All regions significant at $p \leq .001$, uncorrected, $k \geq 10$

Online Resource 13 – Scanner Type and Acquisition Parameters, by Site

	Site 1 - Frankfurt	Site 2 - Aachen	Site 4 - Southampton	Site 7 - Birmingham
Scanner	Siemens Magnetom Tim Trio	Siemens Magnetom Prisma	Siemens Magnetom Tim Trio	Philips Achieva
Software version	Syngo MR A35	Syngo MR D13D	Syngo MR B17	Version 3.2.6.1
Head coil	8-channel	20-channel	32-channel	32-channel
Number of slices	192	192	192	192
Repetition time (TR)	1900ms	1900ms	1900ms	1900ms
Echo time (TE)	2.74ms	3.42ms	4.1ms	3.7ms
Inversion time (TI)	900ms	900ms	900ms	900ms
Field of view	256mm	256mm	256mm	256mm
Flip angle	9º	9º	9º	9º

Online Resource 1~~2~~4 – Flowchart of the Sample Selection Process

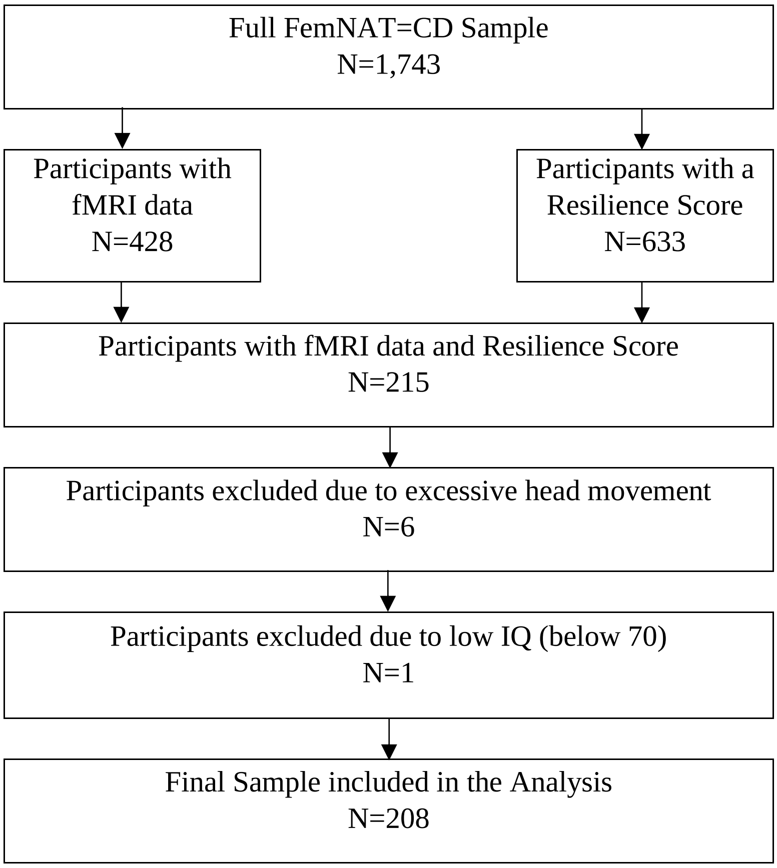

Online Resource 15 – Visual Depiction of the Conscious Face Processing Task

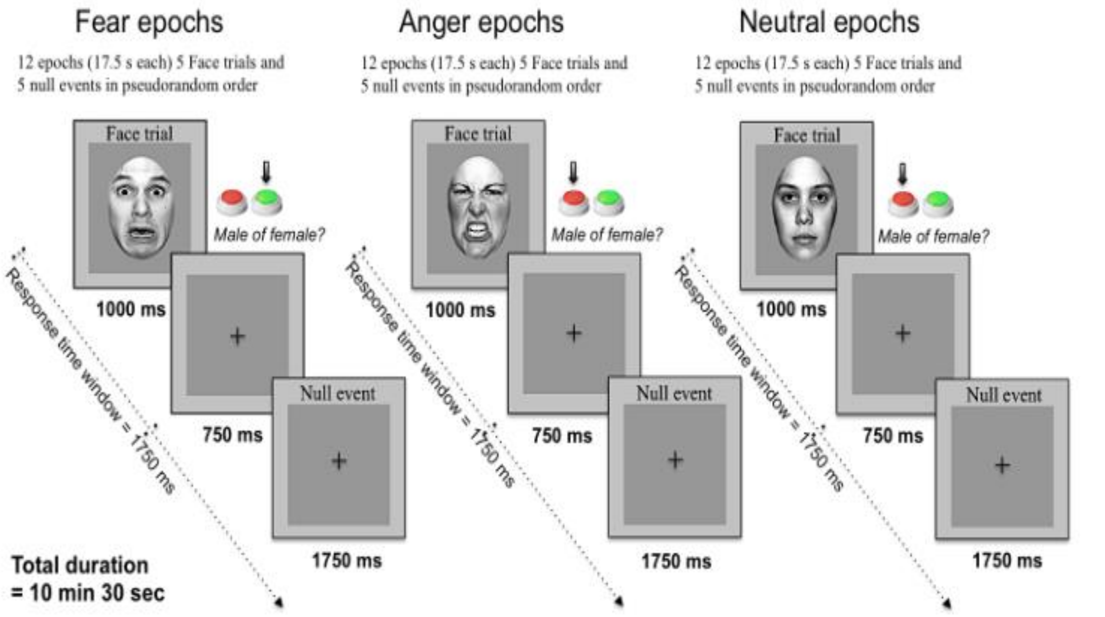

Online Resource 16 – Brain regions, cluster size and coordinates for key contrasts of interest (Anger > Neutral / Fear > Neutral)

					MNI Coordinates		
	Region	Hemisphere	t value	Cluster Size	x	y	z
Anger > Neutral	Inferior Occipital	Right	7.90	432	42	-75	-6
	Inferior Occipital	Left	6.79	97	-30	-87	-3
	Inferior Frontal	Right	6.51	36	-39	-51	-12
Fear > Neutral	Fusiform gyrus	Right	9.18	1061	27	-69	-9
	Middle Temporal	Right	6.15	74	57	-45	9
	Inferior Temporal	Right	6.10	15	42	-42	-15
